# Spontaneous regression of mantle cell lymphoma: a report of four cases

**DOI:** 10.1186/s40880-018-0306-z

**Published:** 2018-05-29

**Authors:** Haige Ye, Aakash Desai, Tiejun Gong, Dongfeng Zeng, Krystle Nomie, Wendy Chen, Wei Wang, Jorge Romaguera, Michael L. Wang

**Affiliations:** 1 Department of Hematology, The First Affiliated Hospital of Wenzhou Medical University, Wenzhou, 325000 Zhejiang P. R. China; 2 Department of Lymphoma/Myeloma, The University of Texas MD Anderson Cancer Center, 1515 Holcombe Blvd, Houston, TX 77030 USA; 3 The University of Texas Health Science Center, Houston, TX 77030 USA; 4 Institute of Hematology and Oncology, Harbin First Hospital, Harbin, 150000 Heilongjiang P. R. China; 5 Department of Hematology, Da Ping Hospital, Research Institute of Surgery, Chongqing Third Military Medical University, Chongqing, 404100 P. R. China

**Keywords:** Mantle cell lymphoma, Spontaneous regression

## Abstract

**Background:**

Spontaneous regression has been reported in some indolent forms of lymphoma. Mantle cell lymphoma (MCL) is an aggressive lymphoid neoplasm and has a poor prognosis. However, approximately 30% of MCL patients can exhibit indolent clinical behavior. To date, complete spontaneous regression of MCL has not been reported.

**Case presentation:**

We describe four cases of spontaneous regression of MCL. At the time of presentation, these patients were asymptomatic, with lymph node enlargement and mild to moderate fluorodeoxyglucose (FDG) uptake on FDG-positron emission tomography combined with computed tomography. One of the possible mechanisms of spontaneous regression of the tumor could be due to the host immune response through humoral and cellular immunity, which may have a role in the clearance of tumor cells.

**Conclusions:**

In this report, we support the use of a “wait and watch” strategy for MCL patients with no risk factors and indolent behavior. This strategy helps spare patients from further potentially harmful chemotherapy. In addition, we describe the phenomenon of spontaneous regression in MCL patients who are asymptomatic and have low-volume disease.

## Background

Patients with various neoplastic diseases have experienced spontaneous regression (SR), including 10%–20% of patients with indolent lymphoma [1]. Compared with SR rates in indolent lymphoma, SR rates in aggressive lymphomas are relatively low [2]. Typically, mantle cell lymphoma (MCL) is an aggressive lymphoid neoplasm and has a poor prognosis. However, approximately 30% of MCL patients exhibit indolent clinical behavior [3].

Despite the indolent clinical behavior in some MCL cases, complete SR of MCL has not been reported, and partial SR of MCL has been reported only once to date. In that report, Kumar et al. [4] described a case of MCL, monitored by serial fluorodeoxyglucose (FDG)-positron emission tomography combined with computed tomography (PET/CT), in which a significant spontaneous reduction in FDG uptake was observed, suggestive of partial regression. Here, we describe four cases of SR of MCL that were observed after a “wait and watch” treatment strategy.

## Case presentation

### Case 1

A 48-year-old man was diagnosed with MCL in July 2010 from an excisional biopsy of the right inguinal lymph node. The pathology was reported as classic MCL, with a Ki-67 value of 10%–15%. Bone marrow biopsy revealed less than 5% involvement, and marrow aspirate flow cytometry showed a small population of phenotypically aberrant CD5-positive B cells monotypic for lambda light chain. At the time of presentation, the patient’s mantle cell international prognostic index (MIPI) score was 2 (one point for serum lactate dehydrogenase and one point for white blood cell count).

**Fig. 1:**
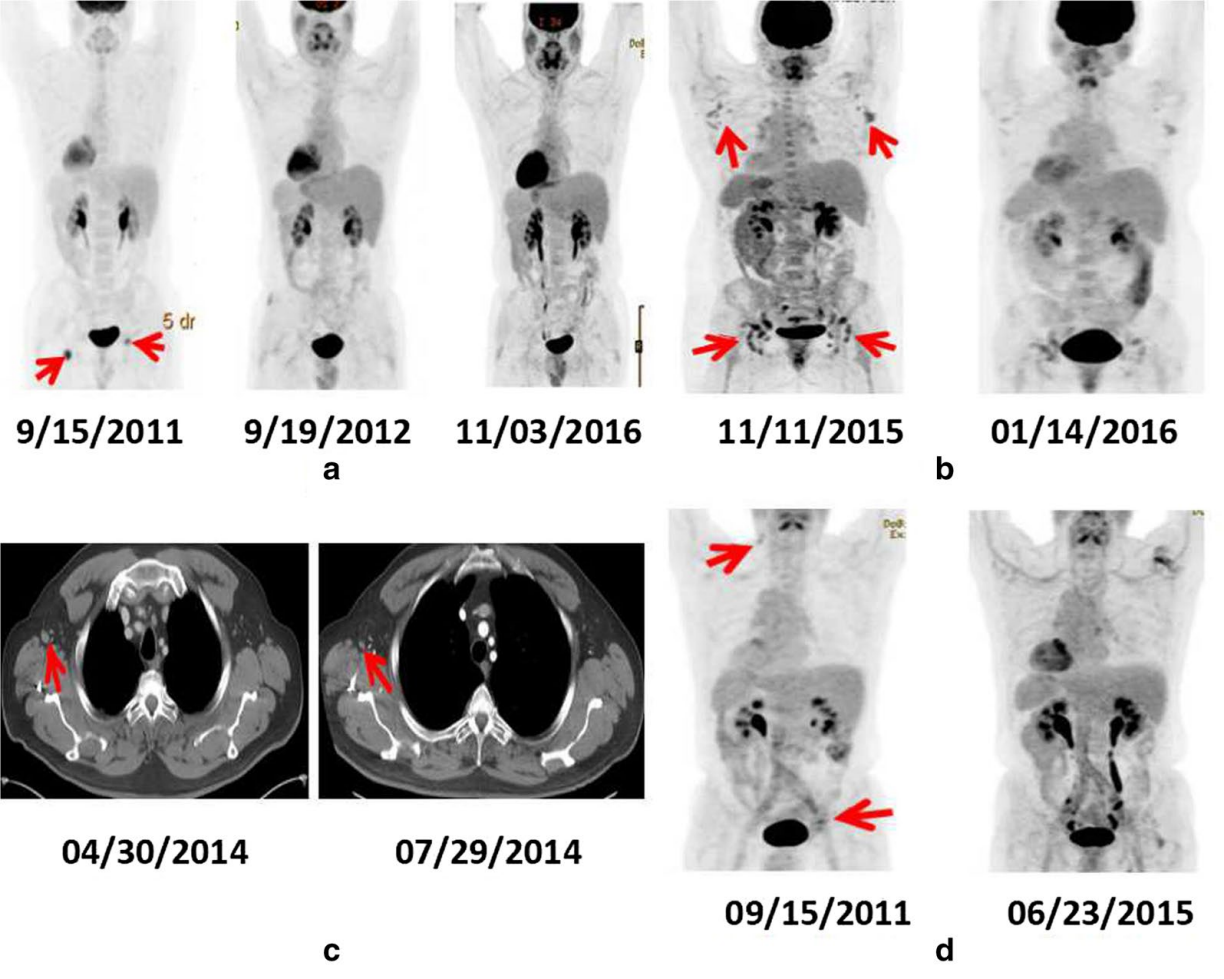
Spontaneous regression of four cases of mantle cell lymphoma. **a** Comparison of positron emission tomography combined with computed tomography (PET/CT) images on September 15, 2011, September 19, 2012, and November 3, 2016, showing the resolution of fluorodeoxyglucose (FDG)-avid lymph nodes in a 48-year-old man with MCL (case 1). **b** Comparison of PET/CT on November 11, 2015, and January 14, 2016, showing the resolution of FDG uptake in the right axillary, prevascular, left obturator, and bilateral inguinal lymph nodes in a 44-year-old woman with MCL (case 2). **c** Chest CT with contrast on April 2014 showed right axillary lymph node enlargement (1.3 × 1.1 cm), whereas chest CT on July 2014 showed a decrease in size (0.8 × 1.0 cm) in a 63-year-old man with MCL (case 3). **d** PET/CT on September 2011 showed low-grade FDG-avid inguinal and left posterior cervical triangle adenopathy (Standardized uptake value was 2.6), whereas PET/CT on June 2015 showed resolution in a 68-year-old man with MCL (case 4). Red arrows indicate FDG-avid lymph nodes

The patient was initially treated with serial monitoring; however, 3 months later, he developed progressive disease and was enrolled in a clinical trial with rituximab, cladribine, and vorinostat, receiving six cycles of chemotherapy between September 2010 and February 2011. He achieved complete remission and received maintenance therapy with rituximab every 2 months between March 2011 and August 2011. However, a left inguinal lymph node grew shortly thereafter in September 2011, and recurrence of MCL was confirmed by core needle biopsy. The pathology report at the time described a diffuse lymphoid infiltrate of non-blastoid cytology composed of small- to medium-sized lymphoid cells that were cyclin D1-positive. Bone marrow and CT examination were otherwise negative for lymphoma. The patient has since been observed without therapy, and his residual adenopathy spontaneously regressed after the first follow-up examination. The most recent PET/CT performed in November 2016 still showed no evidence of lymphoma (Fig. 1a).

### Case 2

A 44-year-old woman with left groin pain for 3–4 weeks in August 2015 underwent CT scans of the abdomen and pelvis, which revealed bilateral inguinal as well as pelvic lymphadenopathy. An ultrasound of the inguinal lymph nodes demonstrated multiple enlarged hypoechoic lymph nodes, the largest measuring 2.7 × 1.3 cm on the left side and 3.3 × 2.4 cm on the right side. A left inguinal lymph node ultrasound-guided needle core biopsy showed MCL with nodular and diffuse patterns and a Ki-67 value of 10%. Fluorescence in situ hybridization (FISH) assays were positive for the translocation between the CCND1 gene located on 11q13 and the IGH gene located on 14q32 (CCND1/IGH) (90% of cells). Later, PET/CT showed mild to moderate FDG uptake in multiple nodal areas including the inguinal, left external iliac chain, and bilateral axillary lymph nodes, with a maximum standardized uptake value (SUV) of 1.3–2.9 (Fig. 1b). Furthermore, there was involvement of the bone marrow with 5% of total bone marrow cellularity.

In October 2015, the patient was referred to MD Anderson Cancer Center, where physical examination revealed palpable bilateral inguinal lymph nodes measuring 1–3 cm. She was asymptomatic with low-volume disease (Table 1) and underwent an observational treatment strategy. A PET/CT in January 2016 showed a decrease in tumor size and uptake of FDG-avid disease (Fig. 1b), suggesting partial SR. In May 2016 and January 2017, repeated PET/CT scans showed stable disease.

**Table 1:** Characteristics of four cases of spontaneous regression of mantle cell lymphoma (MCL)

Characteristic	Case 1	Case 2	Case 3	Case 4
Sex	Male	Female	Male	Male
Age (years)	48	44	63	68
B symptoms	No	No	No	No
LDH/ULN	0.72	0.59	0.62	0.85
β_2_MG	Normal	Normal	Normal	Normal
s-MIPI score	2	1	2	2
Ki-67 value	10%–15%^a^	10%	10%–15%^a^	5%
Pathology pattern	Nodular^b^	Nodular and diffuse	Diffuse^b^	Mantle zone
Involved site (s)	Inguinal	Multiple	Right axillary	Inguinal, cervical
SUV of PET/CT	5.9–8.8	1.3–2.9	NA	2.6
Size of mass	2.5 × 1.4 cm	1.0 × 3.0 cm	1.3 × 1.1 cm	Sub-centimeter
SR	Complete	Partial	Complete	Complete
Time to SR (months)	12	5	3	53
Duration of SR^c^ (months)	18	14	33	20

*LDH/ULN* lactate dehydrogenase/upper limit normal, *β*_*2*_*MG* β_2_ microglobulin, *s-MIPI* simplified MCL-International Prognostic Index, *SUV* standardized uptake value, *PET/CT* positron emission tomography combined with computed tomography, *SR* spontaneous regression, *NA* not available

^a^ Initial Ki-67 value

^b^ Initial pathology pattern, not after relapse

^c^ Still in SR at last follow-up

### Case 3

A 63-year-old man was diagnosed with MCL in October 2009. The initial pathology was reported as a diffuse pattern with a Ki-67 value of 10%–15% and 5% marrow involvement. He was initially treated with rituximab and hyper-CVAD (fractionated cyclophosphamide, vincristine, doxorubicin, and dexamethasone) alternated with rituximab, methotrexate, and cytarabine. In total, the patient received six cycles (three of each) of therapy, which were completed in December 2010, and he achieved complete remission, which lasted for 4 years; in April 2014, however, his disease relapsed in the right axillary lymph node, as evidenced by lymph node biopsy (Fig. 1c). At that time, CT scans with contrast of the neck/abdomen/pelvis and bilateral bone marrow biopsies were all negative for the presence of disease. The patient was then observed without therapy, and 3 months later, chest CT with contrast showed a spontaneous decrease in the size of the lymph node (Fig. 1c). Serial monitoring with CT scans showed no change, and PET/CT scans between October 2016 and April 2017 showed no suspicious activity and spontaneous regression of the existing disease.

### Case 4

A 68-year-old man underwent incidental removal of enlarged right inguinal lymph nodes at the time of surgery for iliac artery aneurysm in January 2011. The pathology was reported as MCL with a mantle zone pattern and with a Ki-67 value of approximately 5%. At that time, the patient was asymptomatic and had no other evidence of lymphoma on CT with contrast of the chest, abdomen, and pelvis. It was decided that he would undergo watchful waiting and serial monitoring of the disease.

In September 2011, a surveillance cervical CT scan with contrast showed sub-centimeter lymph nodes at the left posterior cervical triangle, and PET/CT showed low-grade FDG-avidity at these nodes (Fig. 1d), with an SUV of 2.6 in the left posterior cervical triangle and minimal FDG activity in the bilateral inguinal lymph nodes. Further evaluation at the time included bilateral bone marrow biopsy and upper and lower endoscopic biopsies, both of which showed no evidence of lymphoma.

In June 2015, a follow-up PET/CT showed that the FDG-avid area had regressed spontaneously (Fig. 1d). On the last follow-up in July 2016, the patient showed no signs of MCL.

All four patients denied having had any infections or using steroids during follow-up.

## Discussion

Of the four cases that we describe herein, the regressed lesions of case 1 (relapsed), case 3 (relapsed), and case 2 were confirmed by pathological analysis to be MCL. Although the cervical lesion in case 4 was not confirmed pathologically, we believe that these regressed lymph nodes were highly suspicious for MCL. None of the four patients had a history of ongoing infection, antibiotic use or corticosteroid therapy use for any other diagnosis, any of which could have contributed to disease regression. In addition, no vaccination against any organism was given during the follow-up before regression.

All of the cases presented with good prognostic factors, including low Ki-67 values, low MIPI scores, and non-blastoid cytology. Tumor proliferation is recognized as a strong biological prognostic factor for MCL. Hoster et al. [5] reported differences in time-to-treatment failure and overall survival (OS) between groups with a Ki-67 value of < 30% and ≥ 30% among 543 patients studied. Furthermore, the modified combination of the Ki-67 index and MIPI (MIPI-c) separated 508 patients into four groups with 5-year OS rates of 85, 72, 43, and 17% (*P* < 0.001), and this combination of parameters was more discriminative than MIPI alone [6]. Our cases had a very low simplified MIPI with a Ki-67 value of < 30% (Table 1). In addition, the low SUV in PET/CT has been associated with an indolent clinical process: comparisons between SUVmax > 5 and < 5 in MCL patients have shown that the former group has decreased OS and failure-free survival [7]. Except for case 1, who presented with an SUV > 5 on PET/CT, all cases had an SUV of < 5 on PET/CT.

SR has been frequently reported in indolent lymphomas, including mucosa-associated lymphoid tissue lymphoma [8–11] and low-grade follicular lymphoma [12]. Compared with indolent lymphoma, aggressive non-Hodgkin lymphoma has a relatively low rate of SR [2]. In addition to non-Hodgkin lymphoma, SR has also been reported in classic Hodgkin lymphoma [13]. However, no case of complete SR has been reported in MCL, and only one case of MCL monitored by serial FDG-PET has been reported, which exhibited a significant but partial reduction in FDG uptake on serial whole-body PET scans, suggesting partial regression [4].

As shown in Table 1, the characteristics of the four cases demonstrated indolent behavior and low risk of progression. Interestingly, case 1 progressed after a brief follow-up and required therapy, which is not expected in indolent lymphomas; however, it was later determined that the recurrence had regressed spontaneously.

Possible mechanisms of SR reported in B cell neoplasms include an augmented host immune response through humoral and cellular immunity [2, 14]. For instance, post-allogeneic transplant lymphoproliferative disorders, usually of the aggressive large-cell lymphoma subtype, can be controlled by reducing and/or stopping the immunosuppressing agents [15]. Furthermore, the addition of the immune stimulator interferon-alpha to a doxorubicin-containing regimen for patients with advanced-stage and clinically aggressive follicular lymphomas not only increases progression-free survival but also prolongs OS [16]. The microenvironment in follicular lymphomas has also been reported to play a role in their susceptibility to immune manipulation [17], and recent data support the importance of the microenvironment (which contains immune effector cells) in the survival of MCL cells [18].

The antitumoral response may also be inhibited or stimulated by the reaction of the immune system to the presence of contemporaneous bacteria or viruses or a traumatic intervention [2, 17]. For example, a patient with plasmablastic lymphoma achieved SR after being given antiretroviral therapy against human immunodeficiency virus infection, presumably through the restoration of immune function [19]. In another postulated mechanism of SR in plasmablastic lymphoma, mobilization of the immune system against infection with the Epstein–Barr virus caused tumor regression [20]. A traumatic intervention such as a biopsy can also induce a regional increase in immune system activity and secondary antitumoral activity with subsequent SR [21].

The antitumoral activity of the immune system is believed to be mediated through cytotoxic T lymphocytes [21, 22]. A case report of SR in a patient with diffuse large B-cell lymphoma involving the right breast suggested that CD8-positive T cells might have contributed to the regression [22]. Moreover, in SR of primary cutaneous diffuse large B-cell lymphoma, leg type, immunohistochemical studies demonstrated the presence of lymphocytes positive for CD3, 4, and 8, cytotoxic molecules, granzyme B and T-cell-restricted intracellular antigen (TIA1), both in the vicinity of the tumor nest and in the tumor [21].

A more recent and exciting confirmation of the importance of the immune system, and of T cells in particular, in the treatment of lymphoma is chimeric antigen receptor (CAR) T-cell therapy [23, 24]. Kochenderfer et al. [23] reported that chemotherapy-refractory B-cell malignancies were successfully treated with anti-CD19 CAR T cells. However, data regarding CAR T-cell therapy for MCL are scant. In mouse xenograft models, CAR T cells can be activated by CD19 antigens expressed on the MCL cell surface [25], and MCL cells are sensitive to the CTL019 (cytotoxic T lymphocytes targeting CD19 antigens) effector function. There are currently two ongoing CAR T-cell clinical trials (KITE-C19-102 and KITE-2015-0372) at MD Anderson for the treatment of relapsed or refractory MCL, which have demonstrated promising results thus far (data not published).

## Conclusions

In conclusion, the cases presented in this report support the use of a “wait and watch” strategy for MCL with no risk factors and indolent behavior, such as lack of B symptoms, normal lactate dehydrogenase (LDH) and β_2_ microglobulin (β2M) levels, a low MIPI score, a maximum tumor diameter of less than 3 cm, a low SUV on PET/CT (< 5), a Ki-67 value of < 30%, and non-blastoid cytology. In addition, this report describes the phenomenon of SR in MCL patients who are asymptomatic and have low-volume disease, which can spare patients from further potentially harmful chemotherapy if observed for regression. We also hypothesize that the host immune response through humoral and cellular immunity may play a central role in the clearance of tumor cells and contribute to spontaneous regression.
